# A lack of genetic diversity and minimal adaptive evolutionary divergence in introduced Mysis shrimp after 50 years

**DOI:** 10.1111/eva.13637

**Published:** 2024-01-26

**Authors:** Rebecca G. Cheek, Jessica F. McLaughlin, Maybellene P. Gamboa, Craig A. Marshall, Brett M. Johnson, Douglas B. Silver, Alexander A. Mauro, Cameron K. Ghalambor

**Affiliations:** ^1^ Department of Biology Colorado State University Fort Collins Colorado USA; ^2^ Graduate Degree Program in Ecology Colorado State University Fort Collins Colorado USA; ^3^ Department of Environmental Science, Policy, and Management University of California Berkeley Berkeley California USA; ^4^ Department of Organismal Biology and Ecology Colorado College Colorado Springs Colorado USA; ^5^ Council on Science and Technology Princeton University Princeton New Jersey USA; ^6^ Department of Fish, Wildlife and Conservation Biology Colorado State University Fort Collins Colorado USA; ^7^ Department of Biology, Centre for Biodiversity Dynamics (CBD) Norwegian University of Science and Technology (NTNU) Trondheim Norway

**Keywords:** biological introduction, genotype‐by‐environment association, *Mysis diluviana*, population genetics, rapid evolution

## Abstract

The successes of introduced populations in novel habitats often provide powerful examples of evolution and adaptation. In the 1950s, opossum shrimp (*Mysis diluviana*) individuals from Clearwater Lake in Minnesota, USA were transported and introduced to Twin Lakes in Colorado, USA by fisheries managers to supplement food sources for trout. *Mysis* were subsequently introduced from Twin Lakes into numerous lakes throughout Colorado. Because managers kept detailed records of the timing of the introductions, we had the opportunity to test for evolutionary divergence within a known time interval. Here, we used reduced representation genomic data to investigate patterns of genetic diversity, test for genetic divergence between populations, and for evidence of adaptive evolution within the introduced populations in Colorado. We found very low levels of genetic diversity across all populations, with evidence for some genetic divergence between the Minnesota source population and the introduced populations in Colorado. There was little differentiation among the Colorado populations, consistent with the known provenance of a single founding population, with the exception of the population from Gross Reservoir, Colorado. Demographic modeling suggests that at least one undocumented introduction from an unknown source population hybridized with the population in Gross Reservoir. Despite the overall low genetic diversity we observed, *F*_ST_ outlier and environmental association analyses identified multiple loci exhibiting signatures of selection and adaptive variation related to elevation and lake depth. The success of introduced species is thought to be limited by genetic variation, but our results imply that populations with limited genetic variation can become established in a wide range of novel environments. From an applied perspective, the observed patterns of divergence between populations suggest that genetic analysis can be a useful forensic tool to determine likely sources of invasive species.

## 1 INTRODUCTION

A fundamental goal of evolutionary biology is to understand how populations adapt to novel conditions. Invasive or introduced species are particularly useful in understanding adaptation in nature (Bock et al., 2015), as they are often exposed to a suite of novel environmental conditions to which they are presumed to be initially poorly adapted to (Facon et al., 2006; Prentis et al., 2008). Rapid adaptive evolution as a mechanism for facilitating the establishment of non‐native species in novel environments can explain the success of some introduced species (Bossdorf et al., 2005; Lee, 2002; Marin et al., 2020; Maron et al., 2004; Novak, 2007; Reznick & Ghalambor, 2001). However, adaptive evolution is dependent on genetic variation and how small, introduced populations overcome genetic bottlenecks and drift remains an unresolved question in invasion biology (Estoup et al., 2016).

Studies across multiple taxa have documented the effects of non‐adaptive evolutionary mechanisms such as genetic drift (Puckett et al., 2016) and bottleneck events (Amos & Harwood, 1998; Nei et al., 1975) followed by rapid population recovery (Ortego et al., 2021; Rollins et al., 2015). After initial colonization, many introduced species experience extreme bottlenecks followed by explosive population growth due to ecological release (Ricciardi et al., 2013). The loss of genetic variation via bottlenecks may make species unable to respond to selective processes and impede adaptation (Dlugosch & Parker, 2008; Lande & Shannon, 1996). Yet, a lack of genetic variation does not necessarily impede successful establishment of some invasive species (Estoup et al., 2016; Marin et al., 2020; Schrieber & Lachmuth, 2017). In fact, several studies have demonstrated rapid adaptive evolution in introduced and invasive species despite a loss of genetic variation (Colautti & Lau, 2015; Dlugosch et al., 2015; Schrieber & Lachmuth, 2017; Tsutsui et al., 2000; Willoughby et al., 2018). The selective landscape of novel environments and a small founding population size can even foster adaptation through either demographic founder effects (Szűcs et al., 2017), where adaptive alleles drifting to higher frequency (Tanaka & Wahl, 2022) accelerates the rate of evolution (Schlaepfer et al., 2009; Tsutsui et al., 2000), and/or allowing founding populations to purge deleterious alleles (Facon et al., 2011). This counterintuitive pattern of founding populations with reduced genetic variation adapting to novel environments is known as the genetic paradox of biological invasions (Estoup et al., 2016).

A challenge for many studies of introduced species is a lack of knowledge about the specific source population, the timing of when populations have been established, and evolutionary factors underlying successful establishment (Estoup & Guillemaud, 2010). Our knowledge of introduction pathways for most introduced species relies heavily on direct observation and historical records which are often incomplete or misleading (Estoup & Guillemaud, 2010). Genomic data can play an important role in mediating this knowledge gap by elucidating the genetic composition of introduced populations, thus providing useful information about the history of the invasion process (Dlugosch & Parker, 2008). Genomic data can also identify signatures of adaptive genetic variation and relate them to environmental variation as an indirect method to detect adaptation (Forester et al., 2016, 2018; Hoban et al., 2016; Lotterhos & Whitlock, 2015; Rellstab et al., 2015; Savolainen et al., 2013).

The opossum shrimp, *Mysis diluviana* (formerly M. relicta, Lóven 1862, hereafter *Mysis*), represents an exceptional case for exploring evolutionary implications of human‐directed introductions. This small (<25 mm in body length) stenothermic crustacean is native to cold, deep lakes in the Great Lakes region of the U.S. and central and eastern Canada (Audzijonyte & Väinölä, 2005; Dadswell, 1974). In 1957, fisheries managers transferred a single thermos containing 600–1000 *Mysis* from Clearwater Lake, Minnesota, to Twin Lakes, Colorado with the intention of providing an alternative prey resource for trout (Gregg, 1976; Silver et al., 2021). Limited numbers of individuals were randomly sampled and subsequently transplanted to more than 50 other Colorado water bodies from 1969 to 1975, before their largely negative impacts on recipient ecosystems were understood (Silver et al., 2021). These water bodies encompassed a wide range of environmental conditions (Table 1; Silver et al., 2021). While most Colorado *Mysis* populations were able to persist after just one introduction, some populations required multiple stocking events to become established (Silver et al., 2021). This suggests that some lake environments may have been sufficiently similar to the source environment for populations to become successfully established, while other lake environments were different enough to generate strong selection pressures that hindered the success of some early introduction efforts. We therefore hypothesized that if successful establishment of *Mysis* has been facilitated by weak selection, we should observe little evidence for signatures of selection in the genome and any observed genetic differentiation will reflect non‐adaptive processes. Alternatively, if establishment reflects strong selection and rapid adaptive evolution, genetic‐environment association analyses would result in evidence for adaptive genomic divergence between populations occupying different environments independent of any neutral genetic divergence.

**TABLE 1:** Physiographic and limnological characteristics of the study waters.

Water	Lake depth (m)	Elevation (m.a.s.l)	Area (ha)	Conductivity (ɥS/cm)	Secchi depth (m)	Year(s) stocked	Date sampled	Number stocking events	*N*_indiv_ genotyped
Clearwater Lake	39.6	507	536	48	6.2	Native	08/31/2016	*NA*	23
Twin Lakes	29.3	2804	742	67	4.3	1957	09/25/2014	1	16
Grand Lake	80.8	2550	208	23.3	2.8	1969–1971	08/22/2014	3	20
Carter Lake	54.9	1757	449	71.6	3.1	*NA*	08/23/2017	0	20
Dillon Reservoir	57.3	2749	1276	182	3.0	1970	07/03/2018	2	20
Ruedi Reservoir	78.0	2367	445	100	1.2	1970	06/23/2014	3	20
Gross Reservoir	85.3	2220	167	71.8	5.4	1971–1974	08/28/2019	4	29
Jefferson Reservoir	52.8	3259	45	67.5	6.7	1972	06/07/2018	1	20

*Note*: All the Colorado waters were stocked except Carter Lake which was invaded from an upstream water sometime before 1981 (Nesler, 1986). Maximum lake depth (meters), Elevation (m.a.s.l = meters above sea level), Conductivity (microsiemens per centimeter), and Secchi Depth (meters) of the Minnesota (Clearwater) and Colorado (Twin Lakes) source populations, and focal stocked Colorado populations with the documented year the lakes were stocked (Year Stocked), the date *Mysis* were sampled (day/month/year), the recorded number of stocking events according to Colorado Park and Wildlife records (Silver et al., 2021), and number of individuals genotyped in this study (*N*_indiv_).

*Mysis* have around one generation per year, and successive surveys indicate that introduced *Mysis* populations have similar life histories across their elevational range (see Section 2.1; Silver et al., 2021). *Mysis* have therefore experienced approximately 60 generations since their introduction to Colorado in 1957 to when they were sampled, providing potentially sufficient time to observe evidence for or against adaptive evolution. Previous studies have shown *Mysis* to be impacted by water depth (Schoen et al., 2015), light (Boscarino et al., 2010), and temperature (Berrill & Lasenby, 1983; Dadswell, 1974; Degraeve & Reynolds, 1975) and exhibit poor tolerance to low oxygen environments (Horppila et al., 2003; Sherman et al., 1987). Furthermore, *Mysis* undergo a daily vertical migration from the bottom of the water column to the top at night to feed (Beeton & Bowers, 1982). Therefore, we expect that temperature and oxygen saturation to be important sources of selection on *Mysis* survival in introduced habitats as both these factors are critical for the metabolism of all aerobic aquatic organisms (Wetzel, 2001). Given the documented history of *Mysis* introductions in Colorado and the variation in physiographic and limnological characteristics of the study waters (Table 1), we can explore how patterns of genetic variation inform the evolution of non‐native species to introduced ranges.

**FIGURE 1:**
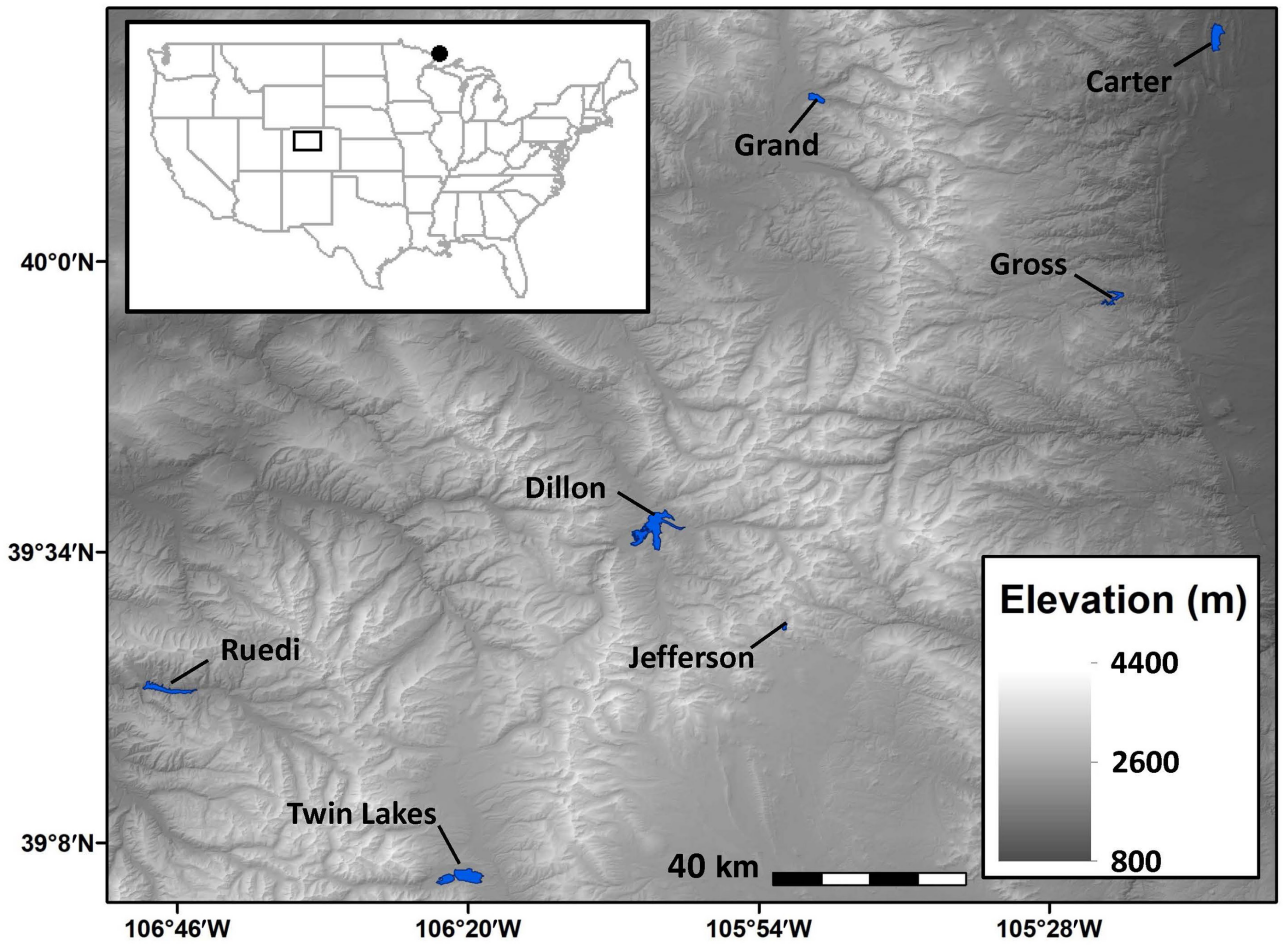
Study area of the southern Rocky Mountains of Colorado, USA. Sampled lakes are shaded in blue and labeled with the lake name and elevation in meters. Genomic analyses of the 168 individuals that passed our quality control thresholds included the source population of *Mysis* from Clearwater Lake, Minnesota (black dot, inset; elevation = 507 m; *n* = 23), and the Colorado source population from Twin Lakes (*n* = 16). Six stocked populations of *Mysis* included individuals from Ruedi Reservoir (*n* = 20), Dillon Reservoir (*n* = 20), Jefferson Reservoir (*n* = 20), Grand Lake (*n* = 20), Carter Lake (*n* = 20), and Gross Reservoir (*n* = 29).

We used Restriction‐site‐Associated DNA sequencing (RADseq) to test if populations of *Mysis* introduced to Colorado exhibit evidence for neutral divergence and putative rapid local adaptation to new environments. Given that *Mysis* have large genomes (Jeffery & Gregory, 2014), our goal is to broadly explore *Mysis* population structure and evidence of selection by addressing two primary research questions. (1) How much genetic variation exists and how much divergence has occurred among introduced populations and between introduced populations and the original source population? (2) Is there evidence of rapid adaptation in introduced populations of *Mysis*? To address these questions, we genotyped individuals from the native source population (Clearwater Lake, Minnesota) and multiple Colorado lakes including the Colorado source (Twin Lakes, Colorado, Figure 1). We then characterized neutral genetic structure within populations of *Mysis* to estimate genome‐wide population divergence and infer the impacts of drift in this system (De Meester et al., 2002; Trumbo et al., 2016). Next, we tested alternative demographic models to determine if undocumented introductions may have taken place as an alternative hypothesis contributing to the spread of *Mysis*. Finally, we used univariate *F*_ST_ outlier and multivariate genotype–environment association (GEA) methods to test for signatures of selection and selection associated with environmental characteristics.

## 2 METHODS

### 2.1 Sample collection

We sampled *Mysis* from the original source population in Clearwater Lake, Minnesota in 2016, the Colorado source population in Twin Lakes in 2014, and six introduced Colorado focal populations from 2014 to 2019 (Figure 1; Table 1). Conductivity and Secchi depth were measured at 3–5 stations, and *Mysis* were collected at 5–10 stations, depending on lake area (Silver et al., 2021). *Mysis* sampling occurred at least 60 min after sundown on moonless nights. We performed one vertical tow with a 1‐m diameter, 500‐μm mesh conical plankton net hauled at 0.4 m/s from the bottom to the surface at each station (Silver et al., 2016). The catch from each haul was preserved in 70% ethanol.

### 2.2 Genotyping

High‐quality genomic DNA was extracted from adult gill tissue using a QIAGEN DNeasy Blood & Tissue kit following the manufacturer's protocol (QIAGEN Inc.). DNA concentration of each sample was quantified using a Qubit fluorometer (Thermo Fischer Scientific). Individual samples that had sufficient DNA yield (>50 ng/μL) were then sent to Admera Health BiPharma Services for ddRAD‐seq library preparation and sequencing.

We used the STACKS version 2.64 *process_radtags* (Catchen et al., 2013; Rochette et al., 2019; Rochette & Catchen, 2017) to demultiplex and filter raw ddRADseq reads for read quality and adapter contamination, remove reads with an uncalled base or low‐quality scores, and rescue barcodes and cutsites with at most one mismatch. We used the *clone_filter* program from STACKS to remove PCR duplicates using a 16 bp random oligo, and *cutadapt* v.1.16 (Martin, 2011) to remove the 10 bp degenerative barcode sequences. In the absence of a closely related reference genome for *Mysis*, we assembled reads de novo using the denovo_map.pl pipeline in STACKS to create an SNP catalog and genotype individuals for individual‐based and population‐based genomic analyses. We ran the pipeline multiple times to optimize the different parameter settings by evaluating how changing the different parameters affected the number of loci following the recommendations of Paris et al. (2017).

We exported the SNP matrix with the *populations* program in STACKS (Catchen et al., 2013), retaining a single random SNP per locus, SNPs with a minor allele count of 2, and present in at least 30% of individuals by population. The resulting SNP matrix was visualized and evaluated for missingness and genotype miscall rates using the R packages, GENOSCAPERTOOLS (Anderson, 2020a; https://github.com/eriqande/genoscapeRtools) and WHOA (Anderson, 2020b; https://github.com/eriqande/whoa). Based on this assessment, we applied additional filters using PLINK 1.90b6.21 (Purcell et al., 2007) by removing loci with a minor allele count of less than three, missingness thresholds of 60% per individual and 50% per variant and Hardy–Weinberg equilibrium exact test *p*‐values below 1e−50. We imputed remaining missing genotype values using a *k*‐nearest neighbor method based on linkage disequilibrium (LD‐kNNi) with the software LINKIMPUTE v1.1.4 (Money et al., 2015). We used this imputed dataset for redundancy analysis (RDA), principal components analysis, discriminant analysis of principle components, and estimates of effective population size (below).

### 2.3 Quantifying intra‐ and inter‐population genetic divergence

We quantified genetic variation and tested for neutral genetic divergence between our sampled populations using a variety of methods. First, we re‐ran *populations* of the catalog from STACKS with a whitelist of SNPs with a minimum allele count of 1 (MAC = 1) and a missingness threshold of 10% per site to output unbiased estimates of π and observed and expected heterozygosity (*H*_obs_ and *H*_exp_), following the recommendations of Schmidt et al. (2021). We ran SNMF in the LEA R package (Frichot et al., 2014; Frichot & François, 2014) in R version 4.3.0 (R Core Team, 2023) using our full filtered dataset to identify potential genetic clusters. Because there are eight lakes, we conducted runs for *K* = 1–9 with ten replicates per *K* value and retained the run with the smallest cross‐entropy to choose the optimal number of clusters. The best supported value of *K* was the estimate for which the cross‐entropy curve exhibited a plateau or a clear minimum value.

To avoid confounding demographic patterns with patterns generated by loci under selection (Luikart et al., 2003), we identified potential candidate loci using two different methods. First, we used the program PCADAPT (Luu et al., 2016) to identify loci showing strong signatures of selection relative to neutral background genomic variation. This methodology controls for population structure by selecting the number of PCs that best summarize the structure in the genetic dataset and has been found to be effective at identifying loci under selection with relatively low false discovery rates compared to other *F*_ST_ outlier tests (Luu et al., 2016). We ran PCADAPT using a false discovery rate of 10% with a *K* = 1 based on visual inspection of a scree plot from our SNMF results testing for optimal *K* between 1 and 9 (Figure S1a). Second, we identified loci strongly associated with different environmental variables of interest using RDA (see Section 2.4). We removed the candidate loci identified by PCADAPT and RDA for downstream neutral population genetics analyses.

We calculated population genetic differentiation (*F*_ST_) using the *populations* program in STACKS with a whitelist of SNPs that passed previous filters with putatively adaptive SNPs identified by PCADAPT and RDA removed. Effective population size (*N*_e_) was estimated using the linkage disequilibrium method in STRATAG (Archer et al., 2016) following Waples et al. (2016). We inferred admixture using the program ADMIXTURE v. 1.3.0 (Alexander et al., 2009) which applies the cross‐validation method to identify the number of PCs for estimating the optimal number of genetic clusters (*K*). We tested values of *K* ranging from 1 to 9 to infer the number of genetic clusters and estimate ancestry within the sampled lakes. The best supported value of *K* was the estimate for which the cross‐entropy curve exhibited a plateau or a clear minimum value. We then used principal components analysis (PCA) implemented in the VEGAN R Package (Oksanen et al., 2020) to visualize how genetic variation is distributed across a reduced number of orthogonal axes without an underlying assumption of genetic groups or spatial structure among sampled lakes. Finally, we used discriminant analysis of principal components (DAPC) using the R package ADEGENET (Jombart, 2008) to maximize the differences between assigned genetic groups to identify potential genetic clusters (Jombart & Ahmed, 2010). Several studies have found that missing data may impact population delineation (e.g. Arnold et al., 2013; Wright et al., 2019; Yi & Latch, 2022). Therefore, we repeated our PCA and DAPC analyses after filtering our dataset to remove SNPs with more than 20% missing data to explore how missing data affects population structure.

**FIGURE 2:**
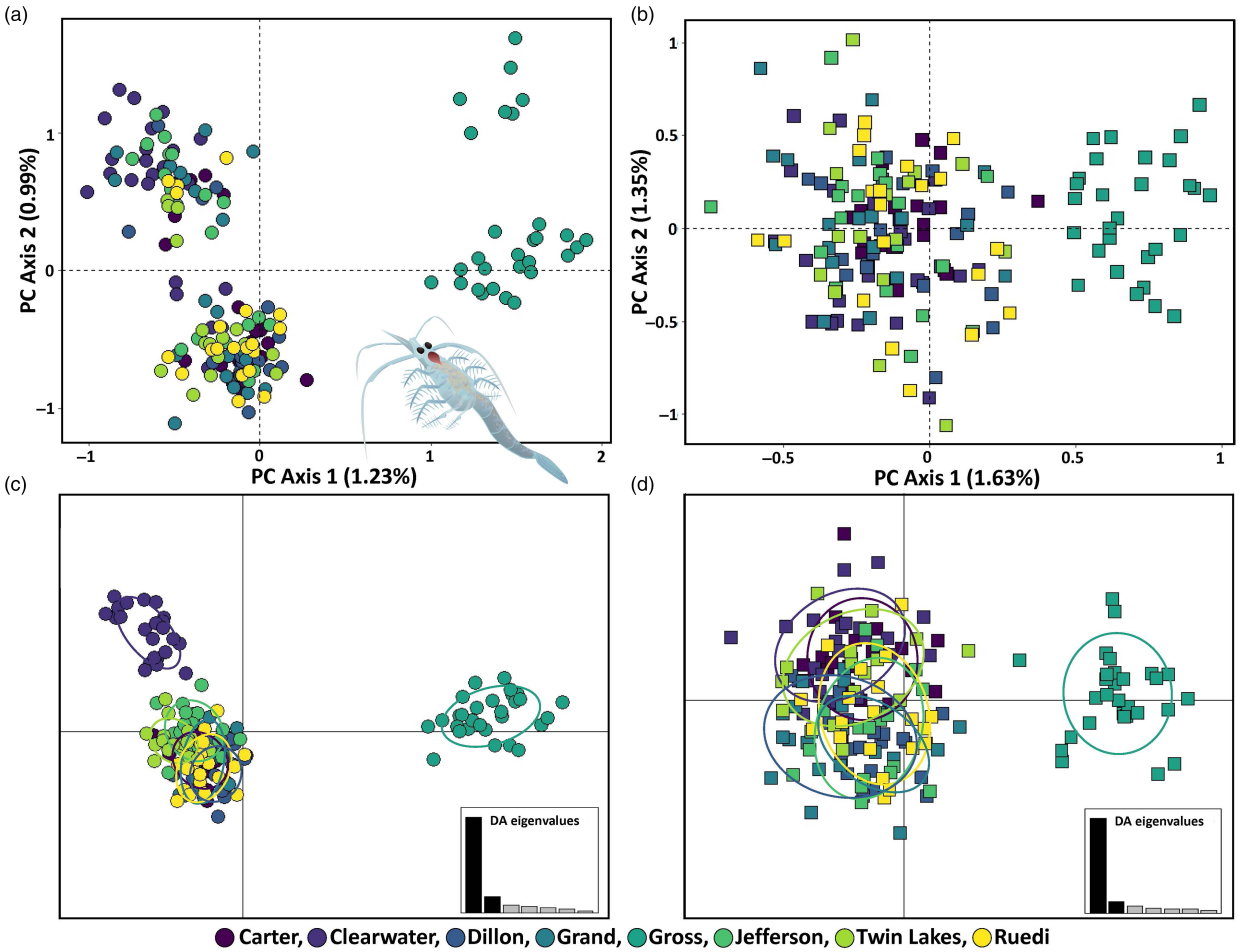
Principal component analyses (PCA) of 168 *Mysis* and 18,220 imputed neutral SNPs (a) show individuals from Gross Reservoir clustering on PC1, while PC2 shows some divergence within, but not between remaining populations. Colors represent the lake from which the *Mysis* was sampled. The finding that the Gross Reservoir is distinct from all other sampled populations was consistent after restricting the dataset to 1371 SNPs with less than 20% missing data according to PCA (b). Discriminant analysis of principal components (DAPC) using 73 PCs, which explained 51.4% of the genetic variation (c), suggests additional structure between the Clearwater Lake source population and all other sampled *Mysis* populations. Repeating the DAPC using 20 PCs, which explained 50.3% of the genetic variation, shows individuals from Gross Reservoir forming a distinct genetic cluster (d). Inset image shows an adult *Mysis diluviana* (illustration by J. F. McLaughlin).

We observed individuals from Gross Reservoir clustering separately from all other populations (Figure 2). To explore this differentiation further, we used Diffusion Analysis for Demographic Inference (∂a∂i; Gutenkunst et al., 2009) to infer the evolutionary relationships among the two main genetic groups identified by PCA. This diffusion approach makes use of the distribution of SNP frequencies to capture haplotype structure and infer recent demographic history (Gutenkunst et al., 2009). Specifically, we tested whether there was evidence to support the current Gross Reservoir population being the result of admixture between a separate, unrecorded introduction and the known introduction from Minnesota that is the source of the other Colorado populations. To do this, we considered the Gross Reservoir individuals as a population and then modeled their relationships with the other Colorado lakes and with the Minnesota source populations separately. If the Gross Reservoir population represents a separate introduction, we expected to find a more ancient split between Gross Reservoir and either of these groups than the known introduction dates, followed by later gene flow consistent with the Colorado introductions.

We converted the filtered imputed vcf file to the specific ∂a∂i input format with easySFS (Overcast, 2017), creating separate input files for Gross Reservoir against the other two populations. These then were tested against a series of models. From the ∂a∂i base models, we tested “neutral” (panmixia), “island” (populations split and grow, with no gene flow), “IM” (populations split and grow, with subsequent gene flow), and “splitmig” (populations split with subsequent gene flow of approximately equal magnitude between populations, but no population growth). To this, we added additional models from McLaughlin et al. (2020): “split_2m” (split with no growth, and migration between each population parameterized separately to detect asymmetric gene flow), “SC_1m” (a split with a discrete period of no gene flow, followed by roughly equal gene flow between populations), and “SC_2m” (as in SC_1m, but with separate migration parameters to detect gene flow asymmetry). Each model was iteratively run until parameter estimates did not approach the bounds of the model space and then was run with those parameters 15 times to ensure the reliability of estimates. The best‐fit model was then selected by calculating AICc from the maximum log composite likelihood score.

### 2.4 Adaptive genetic differentiation

We tested for evidence of selection across our samples using a population‐based RDA (Forester et al., 2016; Rellstab et al., 2015) implemented with the LEA R package (Frichot & François, 2014). As a multivariate GEA analysis, RDA is used to model linear combinations of a single or multiple environmental predictors that maximize the variance explained by linear combinations of the response (e.g. single nucleotide polymorphisms, SNPs; Legendre & Legendre, 2012). RDA has proven to be an effective method at identifying covarying loci strongly associated with environmental predictors with higher true‐positive rates compared to other genotype‐by‐environment tests (Capblancq et al., 2018; Forester et al., 2018). RDA is also highly flexible and robust to variable sample sizes or cases where there is limited population genetic structure (Capblancq & Forester, 2021). We did not have consistent measurements for highly variable environmental factors like water temperature, oxygen saturation, conductivity, or secchi depth for our sampled lakes. Therefore, we identified loci associated with two stable environmental variables for each lake, elevation (meters above sea level, m.a.s.l) and maximum lake depth (meters, m; Table 1), because both are strongly correlated with temperature and dissolved oxygen in freshwater lakes (Novikmec et al., 2013; Woolway et al., 2016). Prior to running the RDA, we confirmed that neither maximum lake depth nor elevation was highly correlated (Pearson's *r* < 0.7). To assess whether selection is contributing to divergence between *Mysis* populations we identified candidate loci from our population‐based RDA based on the “locus score,” which are the coordinates (loading) of each locus in the ordination space. We defined outliers as loci with loading scores greater than 2.5 standard deviations above or below the mean on the two RDA axes to identify SNPs under moderate to strong selection (Forester et al., 2018). We used the absolute value of the predictor with the highest correlation coefficient for each SNP to determine which predictor each outlier locus was most strongly associated with.

We then used an individual‐based PCA to investigate adaptive differentiation between populations using those candidate markers identified by our population‐based RDA and PCADAPT. We only used the candidate markers identified by the RDA and PCADAPT for our individual PCA because we wanted to determine if adaptive divergence could be observed using those markers putatively under selection. Finally, we conducted DAPC of our candidate markers to maximize the differences between assigned genetic groups to identify potential genetic clusters related to selection (Jombart & Ahmed, 2010).

We extracted 350 bp consensus sequences associated with each candidate marker identified by PCADAPT and the RDA. Because we lack a reference genome for *Mysis* or any closely related species, we used the remote option from the Blast+ command line interface of the basic alignment search tool from NCBI (BLAST; McGinnis & Madden, 2004; https://blast.ncbi.nlm.nih.gov/Blast.cgi) to align sequences to potential candidate genes. We used the “nt” database with setting options: max target sequences to 100, expect thresholds to 10 (default), word size to 28 (default), and max matches in a query to 1. We used the R package MYGENE (Mark et al., 2020) to extract accession identifications for each gene found within our query sequences.

## 3 RESULTS

We sequenced 168 individuals from 8 lakes (Figure 1; Table 1). A total of 54,258,265 reads were sequenced with an average of 322,966 paired reads per individual after running *clone_filter*. Of the remaining reads, 92.5% assembled successfully with an average coverage of 10.2× per locus per individual following de‐novo assembly and genotyping in *gstacks*. Following tests of different parameter settings in the *denovo_map* pipeline in STACKS, it appears the sampled *Mysis* are homogeneous as the number of novel SNPs detected in 80% of individuals declined with greater *M* values (the number of mismatches allowed between loci when processing a single individual). At M1, the majority of polymorphism and SNPs were already captured, and the number of polymorphic loci was relatively uniform; the highest polymorphism detected across 80% of the population was at M2. The n setting (number of mismatches allowed between STACKS during construction of the catalog) for *M* + 1 showed the greater number of SNPs (Table S1). We therefore used the parameter settings –*M* = 2, −n = 3. We retained 52,160 SNPs after filtering for a minor allele count of two per locus and present in at least 30% of individuals by population using the *populations* module in STACKS. These SNPs were further filtered in PLINK and 18,441 SNPs passed our missingness thresholds with a final genotyping rate of 49.9% and total missingness of 37.0%. LINKIMPUTE estimated an accuracy of 0.87 for imputed genotypes by randomly masking genotypes. PCADAPT identified seven *F*_ST_ outlier loci, while the RDA detected 215 candidate loci putatively under selection in our imputed dataset (see Section 3.3). One candidate marker was detected by both tests, which resulted in 221 potential candidate loci which were removed for a final dataset of 18, 220 SNPs for neutral population genetic analyses.

### 3.1 Evidence of limited genetic divergence between introduced populations

Measures of genetic diversity (*H*_obs_, *H*_exp_, π), using our matrix of 3803 variant sites following the recommendations from Schmidt et al. (2021), were largely similar for all study sites (Table 2). However, the nucleotide diversity (π) of the Minnesota source population, Clearwater Lake, was highest, while the Dillon Reservoir population had the lowest (Table 2). Neutral population structure estimates using our neutral dataset of 18,220 genetic markers (candidate SNPs removed) revealed differences in effective population size (*N*_e_) between sampled populations (Table 2). The Colorado source population, Twin Lakes, and individuals from Jefferson Lake exhibited the lowest estimates of *N*_e_ (Table 2), whereas the original Minnesota source population, Clearwater Lake, had the highest estimates of *N*_e_. Each sampled population had private alleles, with the greatest number observed in the Gross Reservoir population (Table S2). Pairwise *F*_ST_ estimates between sampling sites were low (Table S3), and our ADMIXTURE analysis of 18,220 unimputed neutral genetic markers indicated *K* = 1 was the best supported value of *K* based on the minimum cross‐validation error (Figure S1a; Table S4; Alexander et al., 2009). Yet PCA identified clear clustering between Gross Reservoir individuals from all other sample populations (Figure 2a; Figure S2). Repeating the PCA with 1371 SNPs with less than 20% missing data and a genotyping rate of 62.9% shows a similar pattern where Gross Reservoir individuals are distinct from all other sampled populations (Figure 2b). These findings align with our DAPC, which identified three main genetic clusters based on discriminant analysis of 73 PCs that explained 51.4% of the total neutral genetic variation using 18,220 SNPs (Figure 2c). Specifically, individuals from the Gross Reservoir population are consistently separated from all other populations. Our DAPC also shows that individuals from the Clearwater Lake source population cluster into a separate group from all other sampled lakes (Figure 2c). However, this clustering of Clearwater Lake individuals is not retained after limiting the dataset to 1371 neutral SNPs with less than 20% missing data based on discriminant analysis of 20PCs which explained 50.3% of the variation (Figure 2d).

**TABLE 2:** Population genomic measures including observed heterozygosity (*H*_obs_), expected heterozygosity (*H*_exp_), and nucleotide diversity (π) across all positions were calculated using a whitelist of 3803 variant sites in the *populations* module in STACKS.

Lake	Polymorphic sites	*H*_obs_	*H*_exp_	π	*N*_e_ (95% CI)
Clearwater Lake	1224	0.05404	0.03939	0.00038	2078 (1892, 2304)
Twin Lakes	937	0.05221	0.03784	0.00036	656 (624, 691)
Grand Lake	1031	0.05231	0.03716	0.00035	1046 (990, 1109)
Carter Lake	1032	0.05309	0.03772	0.00036	1265 (1183, 1361)
Dillon Reservoir	968	0.05117	0.03577	0.00034	1281 (1195, 1380)
Ruedi Reservoir	1079	0.05329	0.03788	0.00036	1921 (1748, 2158)
Gross Reservoir	1104	0.05216	0.03683	0.00035	1038 (1000, 1079)
Jefferson Reservoir	1015	0.05204	0.03699	0.00035	840 (802, 881)

*Note*: Effective population size (*N*_e_) estimates were calculated using 18,220 imputed neutral SNPs with 95% confidence intervals (CI) based on parametric bootstrapping in the STRATAG R package.

### 3.2 Evidence of secondary admixture in gross reservoir

Our demographic models using the full 18,441 imputed SNP dataset found evidence that the Gross Reservoir population is the result of at least two introduction events: the first from an unidentified source population, and the second from the Minnesota source via intermediate transplantation to another Colorado lake. The best‐fit model for the history of the Gross Reservoir population and the combined Colorado populations was secondary contact with a single migration parameter (MLCL = −10,338, AICc = 20,686, AICc weight = 1), with all other models making no contribution to the total weight. We estimated the population size parameter for Gross Reservoir (η_GL_) at 1.54, η_CO_ as 2.75, time of split (*t*_S_) 14.38, time of secondary contact (*t*_SC_) 4.70, and migration (*m*) as 0.13. The optimal value of theta was estimated as 687.92.

### 3.3 Evidence of adaptive evolution to novel habitats

**FIGURE 3:**
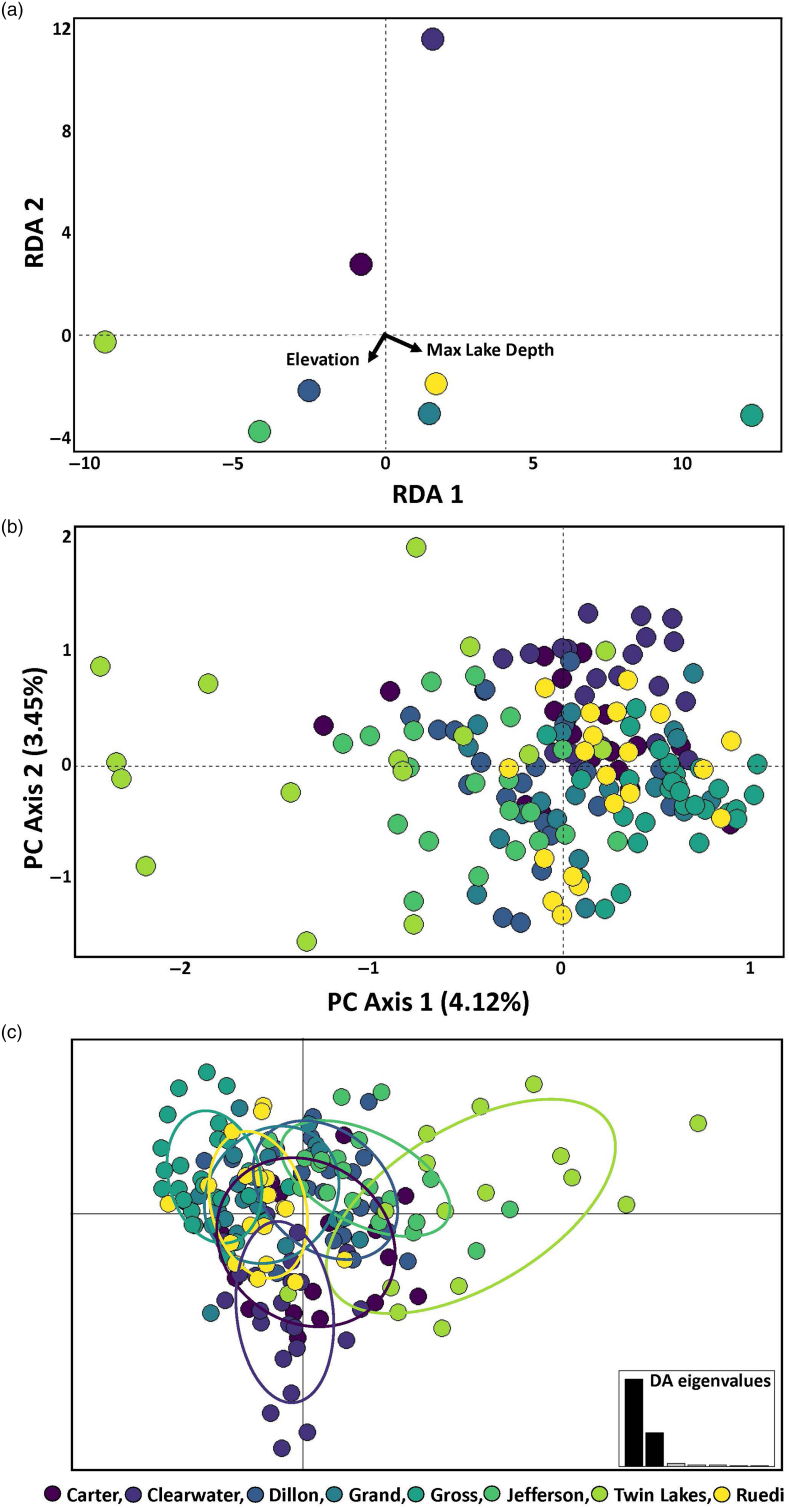
Population‐based redundancy analysis (RDA) using allele frequencies of 168 *Mysis* from eight sampled lakes shows some population‐based genetic association with environment (a). Colored points show where populations from different lakes load on for RDA axes 1 and 2 using 18,441 genetic markers (SNPs) as the response and elevation and maximum lake depth as the predictors (black arrows). Individual‐based PCA (b) using the 221 candidate adaptive markers identified by RDA and PCADAPT shows limited differentiation across adaptive loci. While PC1 and PC2 show some variation between and within populations, there is no clear signature of adaptive divergence between lake populations in the top 10 PC axes. Discriminant analysis of principal components (DAPC) of the 221 candidate adaptive markers using 20 PCs, which explained 50.3% of the genetic variation (c), suggests some additional structure across lakes. Points are shaded by which lake they were sampled matching Figure 2.

*Mysis* populations could be separated by elevation and max lake depth based on population‐based RDA (Figure 3a). We identified 215 candidate loci on our two RDA axes (Figure S3). Slightly more detections were related to max lake depth (120 markers), with 95 candidate loci most strongly correlated with elevation (Figure S3). PCA of the 221 candidate adaptive loci identified by RDA and PCADAPT did not reveal clear adaptive divergence associated with any of our sampled populations in the top ten axes (Figure 3b). However, we observed slight delineation between some lakes in our DAPC using 20 PCs which explained 50.3% of the variation (Figure 3c). We identified only one gene, *zinc finger protein 518A* (Table S5), from one of our 350 bp consensus sequences from our population‐based RDA.

## 4 DISCUSSION

Introduced populations provide an opportunity to evaluate the degree to which evolutionary processes contribute to successful establishment and persistence in new environments. In this study, we analyzed genomic data from introduced populations of *Mysis* in Colorado to test hypotheses related to genetic structure and adaptive genetic differentiation. Using a dataset of 18,441 genetic markers (SNPs), we found that genetic diversity was universally low and that all Colorado populations had relatively fewer polymorphic sites, lower heterozygosity, and nucleotide diversity relative to the original Minnesota source population (Table 2). Consistent with the recorded introduction history of the species in Colorado (Johnson et al., 2018; Silver et al., 2021), our results imply the small founding populations thrived despite overall limited genetic diversity (Dlugosch et al., 2015). We did not find significant population structure between sampled populations. However, Gross Reservoir exhibited greater genetic divergence from all other introduced populations sampled in this study (Figure 2). Surprisingly, we found signals of secondary admixture suggesting an introduction of *Mysis* in Gross Reservoir that was not sourced from either Clearwater or Twin Lakes. We also identified some candidate loci using *F*_ST_ outlier and GEA analyses that warrant further investigation as putative loci under selection. Collectively, our results suggest overall low genetic diversity, evidence of an undocumented introduction event, and some weak evidence to support the hypothesis that rapid adaptation has taken place in introduced *Mysis* populations in Colorado. Below, we discuss these results and how other processes may have contributed to the success of these introduced populations.

### 4.1 Population structure in a human‐introduced freshwater invertebrate

Introduced populations are typically founded by a small number of individuals, resulting in a loss of genetic diversity and reduced potential for adaptive evolution (Frankham, 2005). Yet, paradoxically, many introduced populations can establish and exhibit rapid adaptive evolution (Dlugosch & Parker, 2008). Our findings indicate there is little neutral genetic variation and structure among introduced *Mysis* populations across Colorado and the original Minnesota source population. Compared to studies of other invasive species (e.g. Brookes et al., 2022; Rosenthal et al., 2021), we found low levels of heterozygosity and nucleotide diversity in all sampled populations including the original Clearwater source (Table 2). For example, nucleotide diversity ranged from a high of only 0.00038 in the original source population to a low of 0.00034 in the introduced populations (Table 2). This is consistent with previous genetic work using a limited number of mitochondrial markers which found very little genetic variation across the range of *Mysis diluviana* (Audzijonyte et al., 2005; Audzijonyte & Väinölä, 2005, 2006). Our results are also largely consistent with official records that document a single introduction event into Twin Lakes, Colorado, followed by multiple introductions of *Mysis* to other lakes sourced from Twin Lakes (Silver et al., 2021). However, we cannot exclude the possibility of ongoing gene flow between some of our populations. While *Mysis* are biologically restricted to lakes, they are able to passively disperse to novel water bodies through the use of artificial structures (e.g. pipelines, canals, and tunnels) connected to stocked waters, through natural streams, or hitchhiking on recreational boats (Johnson et al., 2018; Silver et al., 2021). Indeed, one of our sampled Colorado populations, Carter Lake, was colonized by *Mysis* from Grand Lake via a water diversion tunnel that brings water from the western to the eastern side of the North American Continental Divide (Silver et al., 2021). Our pairwise *F*_ST_ values were generally low between lakes, but some of our lowest values between introduced populations were observed between Grand, Jefferson, Carter, and Ruedi Lakes (Table S3). It is difficult to disentangle how much the variation in *F*_ST_ between sampled lakes is due to ongoing gene flow or genetic drift (Holsinger & Weir, 2009; Wright, 1951). However, it would be worthwhile to determine the capacity to which *Mysis* are able to travel between interconnected lakes, which can lead to inadvertent spread and maintenance of *Mysis* populations (Gallardo & Aldridge, 2018; Johnson et al., 2001; Leung et al., 2006).

In addition to low genetic diversity among sampled populations, we did find some weak evidence that *Mysis* went through a bottleneck following their introduction into Colorado. Nucleotide diversity, heterozygosity, and effective population size were highest in the original source population of Clearwater Lake compared to all introduced populations (Table 2). Yet, effective population sizes were relatively higher in some introduced populations, without a clear relationship between nucleotide diversity estimates that would strongly support genetic drift as the sole evolutionary force in this system (Weir & Goudet, 2017; Whitlock & McCauley, 1999). It could be that the interaction between the number of stocking events, or local lake conditions such as lake depth (Schoen et al., 2015), light penetration (Boscarino et al., 2010), temperature (Berrill & Lasenby, 1983; Dadswell, 1974; Degraeve & Reynolds, 1975), or density of zooplankton prey (Caldwell & Wilhelm, 2012; Chess & Stanford, 1998) impact the overall population size and demographics of *Mysis*, which in turn could affect the effective population size (Waples, 2022).

The Gross Reservoir population is notable as it was the only population to show signs of divergence from all other sampled Colorado lakes according to our PCA (Figure 2a,b) and DAPC (Figure 2c,d) analyses. We observed some additional clustering in our PCA along PC axis 2 (Figure 2a), but this is likely driven by sex as we observed this pattern within all lakes and Gross Reservoir. While our SNMF and ADMIXTURE analyses supported a *K* of 1 as the most likely number of clusters, testing a *K* of 2 immediately singled out Gross Reservoir as a separate cluster (Figure S1b). Our demographic modeling strongly supports a hybrid origin for the current *Mysis* population of Gross Reservoir, with contributions from both the Clearwater Lake source (via intermediate introduction to lakes in Colorado) and an unknown source population. The Gross Reservoir population also contained the highest number of private alleles (Table S2). These findings suggest the Gross Reservoir population may be the result of introgression from an alternate source. Yet despite this evidence of introgression, there is still low genetic diversity within Gross Reservoir. It is possible that factors not addressed in this study, such as genetic erosion of the unknown source population (Todesco et al., 2016), could influence the observed genetic diversity in Gross Reservoir.

### 4.2 Rapid adaptation as a mechanism facilitating establishment of introduced populations

Genomic data alone will rarely be definitive in demonstrating local adaptation due to the difficulty associated with the identification of adaptive genetic variation (Hoban et al., 2016; Kardos & Shafer, 2018; Pearse, 2016). Nevertheless, we identified signatures of selection at some loci, and our genomic results show some divergence among introduced populations of *Mysis* from the original source population at loci associated with environmental variation and *F*_ST_ outliers (Figure 3b,c). This evidence indicates weak support for local adaptive differentiation in introduced *Mysis* populations; however, this does not grant a complete picture of potential rapid adaptation in Colorado‐introduced *Mysis*. The *Mysis* transplanted from Clearwater Lake, Minnesota, to Colorado in 1957 evolved at a considerably lower elevation than any of the lakes sampled in this study (Table 1). Our DAPC using only the 221 candidate loci shows the Twin Lakes and Clearwater Lake populations slightly clustering away from each other and all other populations. Therefore, it is possible the initial introduction to Twin Lakes, where all other introduced populations were sourced, imposed a strong enough selective pressure that all other introduced populations had sufficient adaptive genetic variation to become established in high‐elevation lakes in Colorado. Additional experiments measuring physiological traits among introduced populations and the original Clearwater Lake source would help elucidate the potential adaptive traits that may underlie the weak divergence we observe in putative candidate loci.

We cannot exclude the possibility that our reduced representation genomic approach failed to capture most of the *Mysis* genome (Lowry et al., 2017; but see Catchen et al., 2017; Mckinney et al., 2017). *Mysis* have relatively large genomes (>10 pg; Dufresne & Jeffery, 2011; Jeffery & Gregory, 2014). Therefore, it is likely that we sampled only a small fraction of the genome with our reduced representation dataset. While we identified very limited overall genetic diversity (Table 2), it is interesting that we continued to see limited population structure among *Mysis* populations even with a relaxed filter applied to our data (Figure 2). In our attempt to find evidence of local adaptation in *Mysis*, we applied a liberal missingness filter to our data to maximize the number of SNPs retained and increase the likelihood of detecting at least some true positives (Ahrens et al., 2021). Yet, we could only identify weak patterns of differentiation at putative adaptive loci associated with environmental variation. Future studies should further explore these issues by focusing on structural changes such as chromosomal inversions, copy number variants, transposable elements, or gene duplications which have been found to contribute to adaptive evolution in some invasive species (Hoffmann & Weeks, 2007; Makino & Kawata, 2019; Marin et al., 2020; Prevosti et al., 1988). Despite the limitations of RADseq, this study provides a useful overview of genetic variation among introduced *Mysis* populations, and further analyses of additional genomic data may show signals of population differentiation or local adaptation that are undetectable with the limited number of RAD loci presented here.

The lack of a reference genome of *Mysis* or a closely related species, or complementary data on putatively adaptive traits, also hampers our ability to make inferences about genes under selection. A cautious overview of our BLAST search results indicates that one of the sequences containing a candidate genetic marker identified in our RDA occurs within *zinc finger protein 518A* (GenBank accession number XM_034309432) which is linked to cell signaling (Sayers et al., 2022). All other comparative sequences from BLAST did not have annotation information. We detected one SNP that was identified by both PCADAPT and RDA as a potential candidate under selection. As a univariate *F*_ST_ outlier method, PCADAPT is biased toward identifying large‐effect loci undergoing strong selection (Hoban et al., 2016; Wellenreuther & Hansson, 2016). Large‐effect loci beneficial to fitness may respond more strongly to selection and avoid loss during founder events common in introduced or invasive species (Dlugosch et al., 2015; Kimura, 1985). Therefore, the fact that we identified a locus using both univariate PCADAPT and the multivariate RDA suggests that some loci associated with environmental variables may be important to fitness. While there is little evidence of overall differentiation using the adaptive SNPs, it is possible that there are genes that impact the success of *Mysis* that we are unable to detect with our current dataset.

While we assumed that introduced *Mysis* are undergoing strong selective pressure due to the differences between the source and introduction lakes leading to variable success of stocking efforts in some lakes (Silver et al., 2021), it is possible that the environmental predictors we tested are not correlated with selection pressures associated with *Mysis* or our environmental predictors were not measured at an appropriate scale to identify associated SNPs (Forester et al., 2018; Rellstab et al., 2015; Whitlock & Lotterhos, 2015). It is also possible that the Colorado lakes fall within the range of *Mysis* environmental tolerance. *Mysis* species are tolerant of low temperatures (Rudstam et al., 1999) and have dark‐adapted eyes (Feldman et al., 2020), which reflect their circumarctic evolutionary history within cold, deep lakes. Their diel vertical migration behavior exposes them to a wide range of thermal conditions (Rudstam et al., 1999). Thus, it could be that *Mysis* are sufficiently plastic and possess a broader physiological breadth than we currently appreciate (Davidson et al., 2011). This system offers a valuable opportunity to study putative adaptive traits and phenotypic plasticity of replicated populations with a shared genomic background.

### 4.3 M*ysis* and the lasting conservation implications of introductions

The human‐mediated introduction of non‐native species can have profound negative impacts on the ecology of invaded ecosystems (Lodge, 1993; Vitousek et al., 1996, 1997). In the case of *Mysis*, a chronic decline of multiple fish species was observed in multiple lakes in the decades following *Mysis* introduction and establishment (Northcote, 1991). A long history of studies beginning in the 1970s have demonstrated that *Mysis* significantly outcompete fish for zooplankton prey (Finnell, 1977; Lansenby, 1971; Lasenby et al., 1986; Nesler & Bergersen, 1991), which catalyzed the destruction of some local food webs (Ellis et al., 2011; Spencer et al., 1991), eutrophication (Caires et al., 2013), and extirpation of native species in some cases (Devlin et al., 2017; Lasenby et al., 1986). Some mitigation efforts for *Mysis* have been proposed (Ashley et al., 1997; Martinez et al., 2009), and physical removal is occurring in some waters (Schindler et al., 2012; Schladow et al., 2021), but no practical means of eliminating *Mysis* has been found (Fredrickson, 2017).

Like many other invertebrate species introduced in small numbers initially (e.g. O'Grady et al., 2002; Orwig et al., 2002; Tracy & Robbins, 2009), *Mysis* have thrived in their introduced ranges. Several intrinsic factors have likely contributed to the success of *Mysis* introductions. They have relatively short generation times, as females may reach sexual maturity in 1 year in ideal conditions (Caldwell & Wilhelm, 2012), can produce 10–40 offspring annually over a lifespan of 1–3 years (Beeton & Gannon, 1991; Silver et al., 2021), and can occur in high population densities (Jude et al., 2018). These life history traits that promote high fecundity and large population sizes may have enabled *Mysis* populations to become established and persist in a diversity of lake environments.

Our intention is to offer the *Mysis* system as a case study to inform future management efforts. Unlike some introduced or invaded systems (Estoup & Guillemaud, 2010), it is noteworthy that a strength of the *Mysis* system is that we have clear records detailing the introduction history of *Mysis* in Colorado (Silver et al., 2021). Yet the genomic data we present here suggest that at least one undocumented introduction took place in Gross Reservoir. We do not have sufficient data to speculate on the origins of this unknown introduction, but future studies will benefit from testing the accuracy of *Mysis* introduction records by incorporating genetic data from multiple potential sources. Moving forward, coupling genomics with direct observational data and historical records, as we have done here, improves our ability to reconstruct the introduction pathways of introduced and invasive species which is critical in implementing effective management policies (Estoup & Guillemaud, 2010; Johnson et al., 2009; Rosenthal et al., 2021; Smyser et al., 2020; Willson et al., 2011). In cases where historical records are lacking, for instance, when an invasive species is introduced surreptitiously (Johnson et al., 2009), the ability to evaluate potential source populations with genetic analyses could assist with criminal investigations following illegal introductions.

## 5 CONCLUSIONS

Our study highlights the strengths and limitations of genomic data inspecies with limited data available. First, by designing our genetic sampling to include both the original source populations at Clearwater Lake and Twin Lakes and multiple introduced populations in Colorado, we were able to determine that there is little overall genetic variation in sampled populations, which is consistent with repeated introductions from the same source population. Second, we were able to detect signals of introgression in Gross Reservoir *Mysis*, suggesting that undocumented introduction(s) have occurred and possibly facilitated the spread of *Mysis* in Colorado. Third, genomic‐scale data suggest a limited number of loci may be informative for patterns of adaptive variation, but an overall lack of clear adaptive differentiation between introduced populations. However, we cannot rule out some alternative explanations, such as uniform environmental conditions relevant for *Mysis* in the sampled lakes, or inadequate sampling of the *Mysis* genome. Overall, it is clear that the genomic paradox of biological invasion is observed in *Mysis* where limited genetic variation has not hindered the successful establishment of introduced populations.

## CONFLICT OF INTEREST STATEMENT

The authors declare no conflict of interest.

## Supporting information


Data S1

## Data Availability

Filtered VCFs are available on Dryad https://doi.org/10.5061/dryad.8cz8w9gwx. Code and associated data are available at https://github.com/RGCheek/Mysis_Shrimp_genomics.
