# Supplementary material for: A lack of genetic diversity and minimal adaptive evolutionary divergence in introduced Mysis shrimp after 50 years
Source: Evol Appl. 2024 Jan 26;17(1):e13637. doi: 10.1111/eva.13637 (PMC10818135; doi:10.1111/eva.13637)
Supplement: Supplementary file 1 — Data S1 [file EVA-17-e13637-s001.zip › Supplemental_Figures_and_Tables_Revised.docx]

**Supplemental Materials**

**
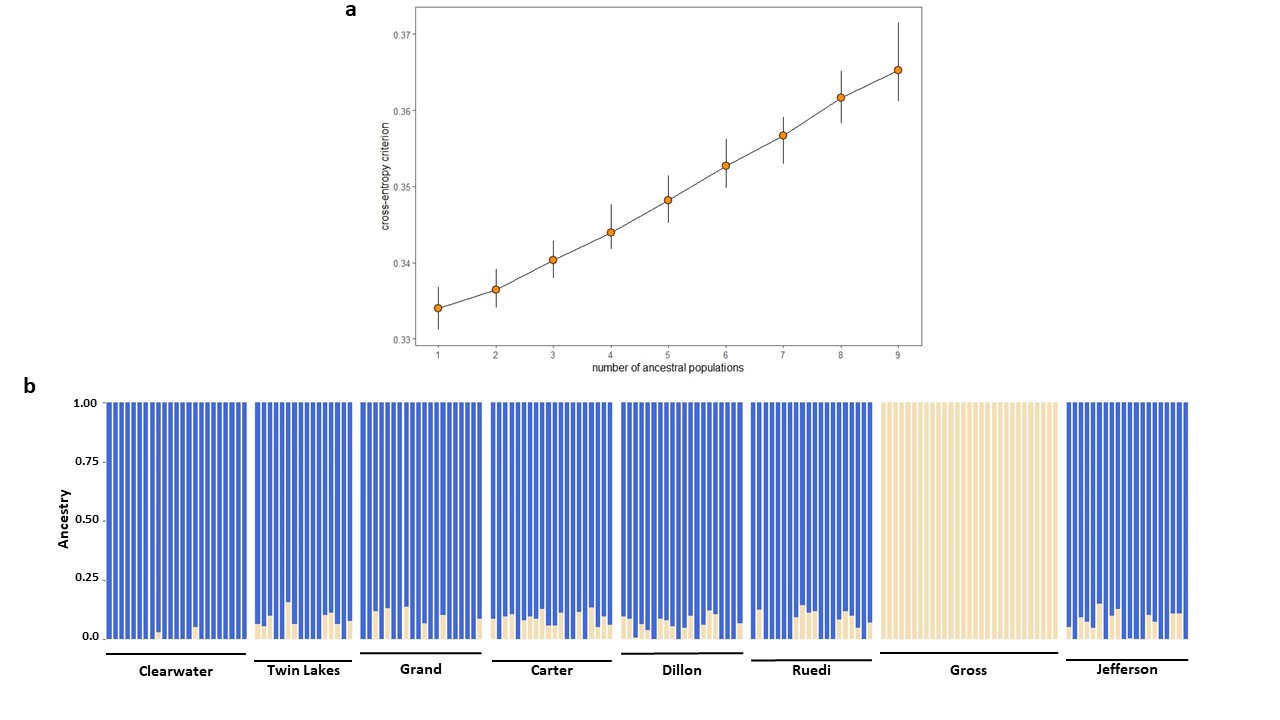
**

**Figure S1**: Population structure analyses of 168 *Mysis diluviana* from the original Minnesota (Clearwater, n=23) and Colorado (Twin Lakes, n=16) source populations and our sampled translocated populations are arranged by year stocked (Grand, n = 20; Carter, n =20; Dillon, n = 20; Ruedi, n=20; Gross, n=29; Jefferson, n=20). Panel a) shows the cross-entropy criterion results from SNMF of all runs of K (K= 1 through 9) using all 18,441 imputed genetic markers (SNPs). Panel b) shows Admixture assignment plots for K=2 using 18,220 non-imputed neutral SNPs and 168 *Mysis* shrimp from the original Minnesota (Clearwater, n=23) and Colorado (Twin Lakes, n=16) source populations identify the Gross Reservoir population as a unique genetic cluster. Sampled introduced populations are arranged by year stocked.


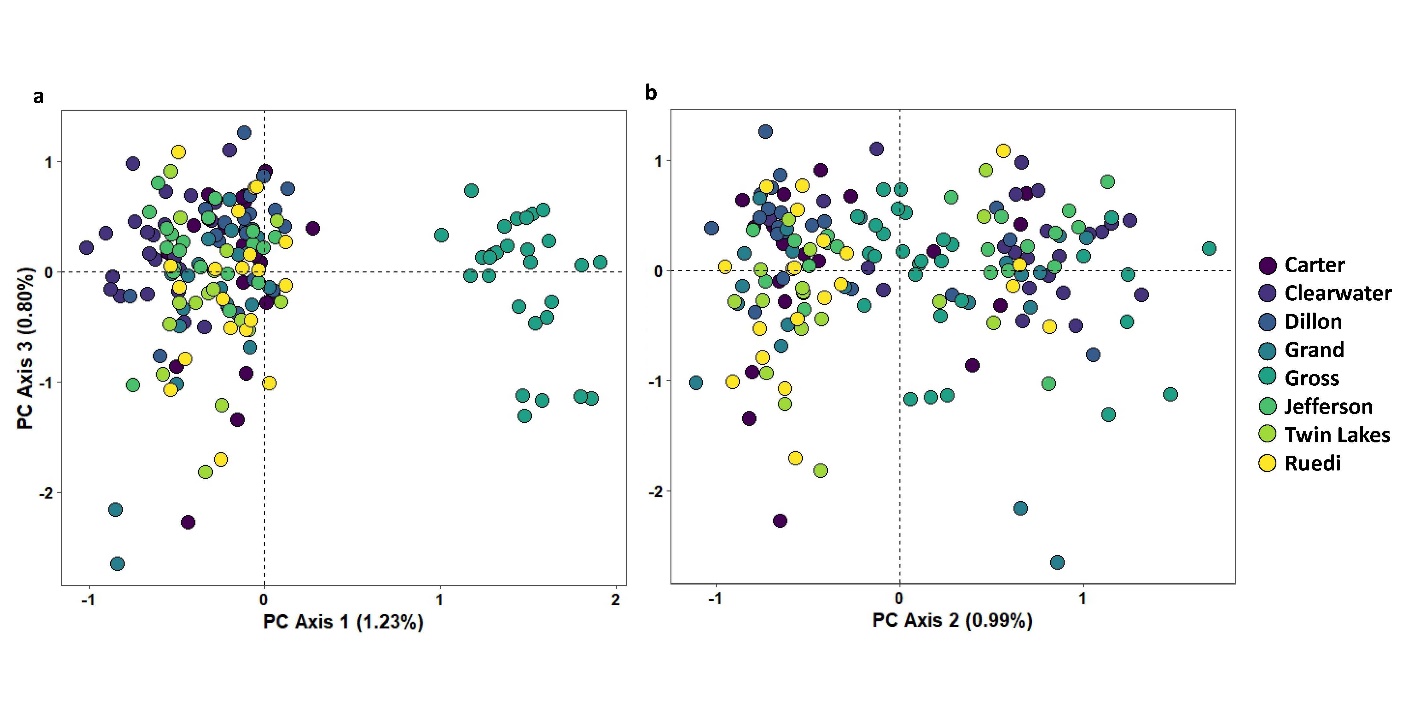


**Figure S2**: Principal component analyses (PCA) of 168 *Mysis* and 18,220 imputed neutral genetic markers (SNPs) shows individuals from Gross Reservoir clustering on PC1 (a) while PC2 and PC3 show little divergence between lake populations. Individuals (colored points) are shaded by sampled lake (b).

**
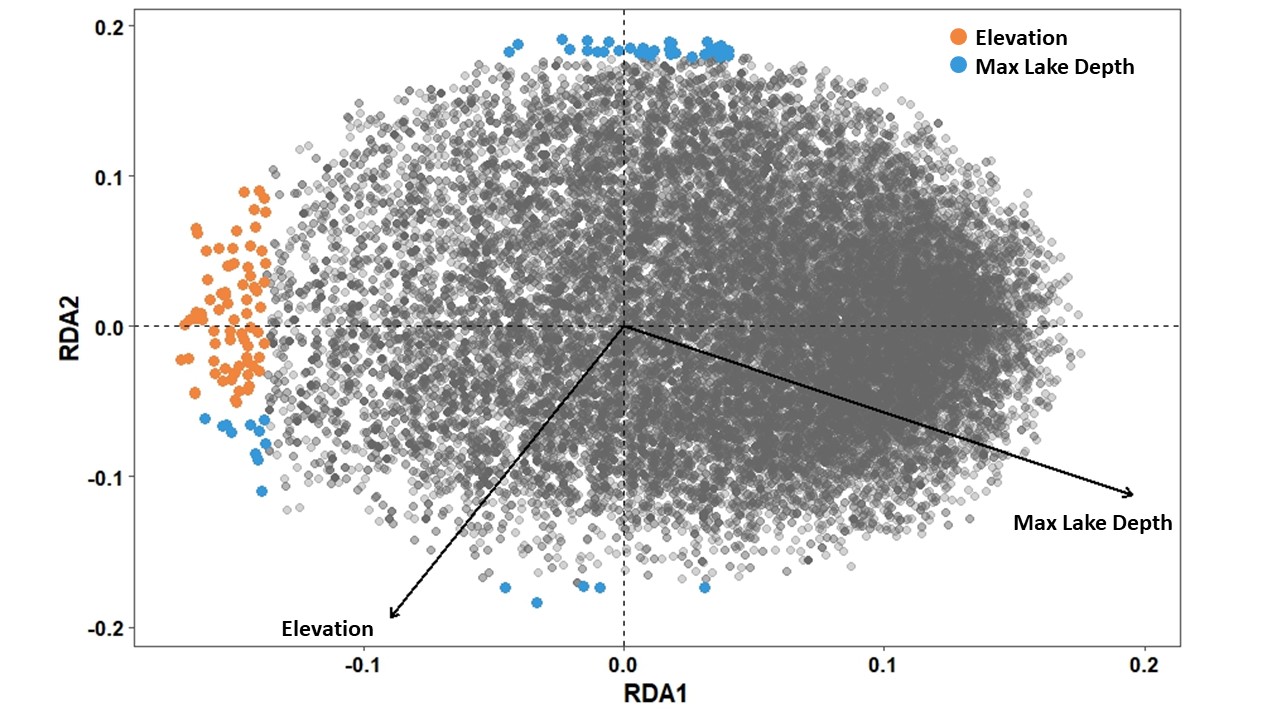
**

**Figure S3:** Population-based redundancy analysis biplot of 18,441 imputed SNPs using lake depth (m) and elevation (m.a.s.l) as the predictors. Colored SNPs were identified as candidates if they were greater than +/- 2.5 standard deviations from the mean loading on RDA axis 1 & 2. SNPs that correlated more strongly with elevation are shown in orange, while SNPs that correlated more strongly with lake depth are shaded blue. Presumably neutral SNPs are coded as grey.

**Table S2:** Genetic diversity of introduced *Mysis* sampled across Colorado as well as the original source population (Clearwater Lake) using a whitelist of 18,220 neutral variant sites in the *populations* module in STACKS.

| **Lake** | **Polymorphic Sites** | **Private Alleles** |
| --- | --- | --- |
| **Clearwater Lake** | 12,999 | 19 |
| **Twin Lakes** | 12,165 | 10 |
| **Grand Lake** | 13,290 | 9 |
| **Carter Lake** | 13,097 | 14 |
| **Dillon Reservoir** | 12,978 | 7 |
| **Ruedi Reservoir** | 13,304 | 10 |
| **Gross Reservoir** | 13,064 | 137 |

**Table S3:** Pairwise F_ST_ estimates using the *populations* module in STACKS and a whitelist of 18,220 neutral genetic markers (SNPs). *Mysis* populations are arranged by the original source population at Clearwater Lake, Minnesota, the Colorado source population at Twin Lakes, and by year stocked for each introduced population.

| **Year Stocked** | **1957** | **1969-1971** | ***Unknown*** | **1970** | **1970** | **1971-1974** | **1972** |
| --- | --- | --- | --- | --- | --- | --- | --- |
| **Population** | **Twin Lakes** | **Grand** | **Carter** | **Dillon** | **Ruedi** | **Gross** | **Jefferson** |
| **Clearwater** | 0.0224 | 0.0199 | 0.0209 | 0.0213 | 0.0204 | 0.0245 | 0.0202 |
| **Twin Lakes** |  | 0.0219 | 0.0228 | 0.0235 | 0.0220 | 0.0258 | 0.0233 |
| **Grand** |  |  | 0.0201 | 0.0206 | 0.0198 | 0.0238 | 0.0198 |
| **Carter** |  |  |  | 0.0209 | 0.0202 | 0.0239 | 0.0210 |
| **Dillon** |  |  |  |  | 0.0207 | 0.0245 | 0.0211 |
| **Ruedi** |  |  |  |  |  | 0.0238 | 0.0204 |
| **Gross** |  |  |  |  |  |  | 0.0240 |

**Table S4:** Cross validation cross-entropy criterion results from ADMIXTURE using 18,220 un-imputed neutral SNPs support a K=1 as the optimal number of clusters.

| Number of clusters (K) | Cross Validation Error |
| --- | --- |
| 1 | 0.31976 |
| 2 | 0.33544 |
| 3 | 0.35894 |
| 4 | 0.38093 |
| 5 | 0.40818 |
| 6 | 0.43413 |
| 7 | 0.45999 |
| 8 | 0.48423 |
| 9 | 0.50919 |
